# Cellular Profiles of Prodynorphin and Preproenkephalin mRNA-Expressing Neurons in the Anterior Olfactory Tubercle of Mice

**DOI:** 10.3389/fncir.2022.908964

**Published:** 2022-07-22

**Authors:** Ayako Maegawa, Koshi Murata, Kazuki Kuroda, Shigeharu Fujieda, Yugo Fukazawa

**Affiliations:** ^1^Department of Otorhinolaryngology-Head and Neck Surgery, Faculty of Medical Sciences, University of Fukui, Fukui, Japan; ^2^Division of Brain Structure and Function, Faculty of Medical Sciences, University of Fukui, Fukui, Japan; ^3^Life Science Innovation Center, Faculty of Medical Science, University of Fukui, Fukui, Japan; ^4^Research Center for Child Mental Health Development, Faculty of Medical Sciences, University of Fukui, Fukui, Japan

**Keywords:** olfactory tubercle, prodynorphin, preproenkephalin, opioids, medium spiny neurons, dopamine receptor D1, dopamine receptor D2, DARPP-32

## Abstract

The olfactory tubercle (OT) is a striatal region that receives olfactory inputs. mRNAs of prodynorphin (Pdyn) and preproenkephalin (Penk), precursors of dynorphins and enkephalins, respectively, are strongly expressed in the striatum. Both produce opioid peptides with various physiological effects such as pain relief and euphoria. Recent studies have revealed that OT has anatomical and cytoarchitectonic domains that play different roles in odor-induced motivated behavior. Neuronal subtypes of the OT can be distinguished by their expression of the dopamine receptors D1 (Drd1) and D2 (Drd2). Here, we addressed whether and which type of opioid peptide precursors the D1- and D2-expressing neurons in the OT express. We used multiple fluorescence *in situ* hybridization for mRNAs of the opioid precursors and dopamine receptors to characterize mouse OT neurons. Pdyn was mainly expressed by Drd1-expressing cells in the dense cell layer (DCL) of the OT, whereas Penk was expressed primarily by Drd2-expressing cells in the DCL. We also confirmed the presence of a larger population of Pdyn-Penk-Drd1 co-expressing cells in the DCL of the anteromedial OT compared with the anterolateral OT. These observations will help understand whether and how dynorphins and enkephalins in the OT are involved in diverse odor-induced motivated behaviors.

## Introduction

The olfactory tubercle (OT) plays an essential role in acquiring odor-induced motivated behavior (Xiong and Wesson, 2016; Yamaguchi, 2017; Murata, 2020). The OT is part of the ventral striatum along with the nucleus accumbens (NAc) and is also referred to as the tubular striatum (Wesson, 2020; Wright and Wesson, 2021). The OT receives substantial dopaminergic inputs from the ventral tegmental area (Ikemoto, 2007; Park et al., 2017; Zhang et al., 2017a; Poulin et al., 2018) as well as olfactory inputs directly from the olfactory bulb and indirectly from the olfactory cortex and prefrontal cortex (Neville and Haberly, 2004; Zhang et al., 2017b).

The principal neurons of the OT comprise three major subtypes: the medium spiny neurons (MSNs), dwarf cells, and granule cells (Millhouse and Heimer, 1984; Murata et al., 2015; Xiong and Wesson, 2016). MSNs are distributed throughout the OT, forming a dense cell layer (DCL, also referred to as layer II). The dwarf cells are clustered in the lateral part of the OT, forming the Cap region, which is interspersed throughout the anteroposterior axis (Hosoya and Hirata, 1974). The granule cells are clustered through the anteromedial surface to the deep layers of the central OT, forming the Islands of Calleja (ICj), which seems to be a continuous structure (Fallon et al., 1978; de Vente et al., 2001).

MSNs, dwarf cells, and granule cells express dopamine receptor subtypes differently: MSNs express dopamine receptor D1 (Drd1) or D2 (Drd2), dwarf cells express Drd1, and granule cells express D3 (Drd3) and weakly Drd1 (Yung et al., 1995; Murata et al., 2015; Zhang et al., 2021). Recent studies have revealed distinct roles of Drd1-, Drd2-, and Drd3-expressing neurons in motivated behaviors. Drd1- or Drd2-expressing MSNs in the anteromedial OT are involved in learned odor-induced attractive or aversive behaviors, respectively (Murata et al., 2015, 2019a; Murofushi et al., 2018). Activation of Drd1-expressing MSNs and dwarf cells in the lateral OT is accompanied by learned odor-induced aversive behavior (Murata et al., 2015). Drd3-expressing granule cells in the ICj have been shown to be involved in grooming behavior (Zhang et al., 2021). Domains of the OT also have distinct roles in motivated behaviors. An intracranial self-administration study, for example, revealed that the anteromedial OT plays a critical role in mediating rewarding action of cocaine compared to other OT regions (Ikemoto, 2003). However, the mechanisms underlying the subtype- and domain-specific roles of OT neurons remain to be elucidated.

Opioids provide pain relief and euphoric effects (Barbano and Cador, 2007; Corder et al., 2018). The opioid receptors are Gi/o-coupled types and have the three major subtypes of mu, delta, and kappa receptors. Their endogenous ligands with strong affinity are endorphins, enkephalins, and dynorphins, respectively (Waldhoer et al., 2004). The precursor genes for dynorphins and enkephalins are prodynorphin (Pdyn) and preproenkephalin (Penk), respectively, both of which are strongly expressed in the striatum (Harlan et al., 1987; Besson et al., 1990; Merchenthaler et al., 1997; Cansler et al., 2020). Curran and Watson conducted an in-depth analysis of the cell type of opioid and dopamine receptor subtype-expressing neurons in the NAc using double *in situ* hybridization (ISH) (Curran and Watson, 1995). They revealed that Pdyn is mainly expressed by Drd1-expressing cells, whereas Penk is expressed primarily by Drd2-expressing cells in the NAc. Although neuronal subtypes of the OT express Pdyn, Penk or both peptides (Furuta et al., 2002), the cellular profiles for the expression of opioid and dopamine receptor subtypes in the OT are unknown. In the present study, we performed triple-fluorescence ISH for opioid and dopamine receptor mRNAs and examined cellular profile of the anterior OT where Drd1- and Drd2-expressing MSNs are differentially involved in attractive and aversive behaviors (Murata et al., 2015). We confirmed the subtype-specific expression of opioids and newly identified a neuronal subtype of Pdyn-Penk-Drd1 co-expressing cells in the DCL of the anterior OT. We also confirmed that the Pdyn-Penk-Drd1 co-expressing cells expressed DARPP-32 (Ouimet et al., 1984), suggesting their neurochemical features of striatal cells.

## Materials and Methods

### Ethical Statements

All experiments were conducted in accordance with the Guidelines for Animal Experimentation in Neuroscience of the Japan Neuroscience Society and were approved by the Experimental Animal Research Committee of the University of Fukui.

### Animals

**Figure 1:**
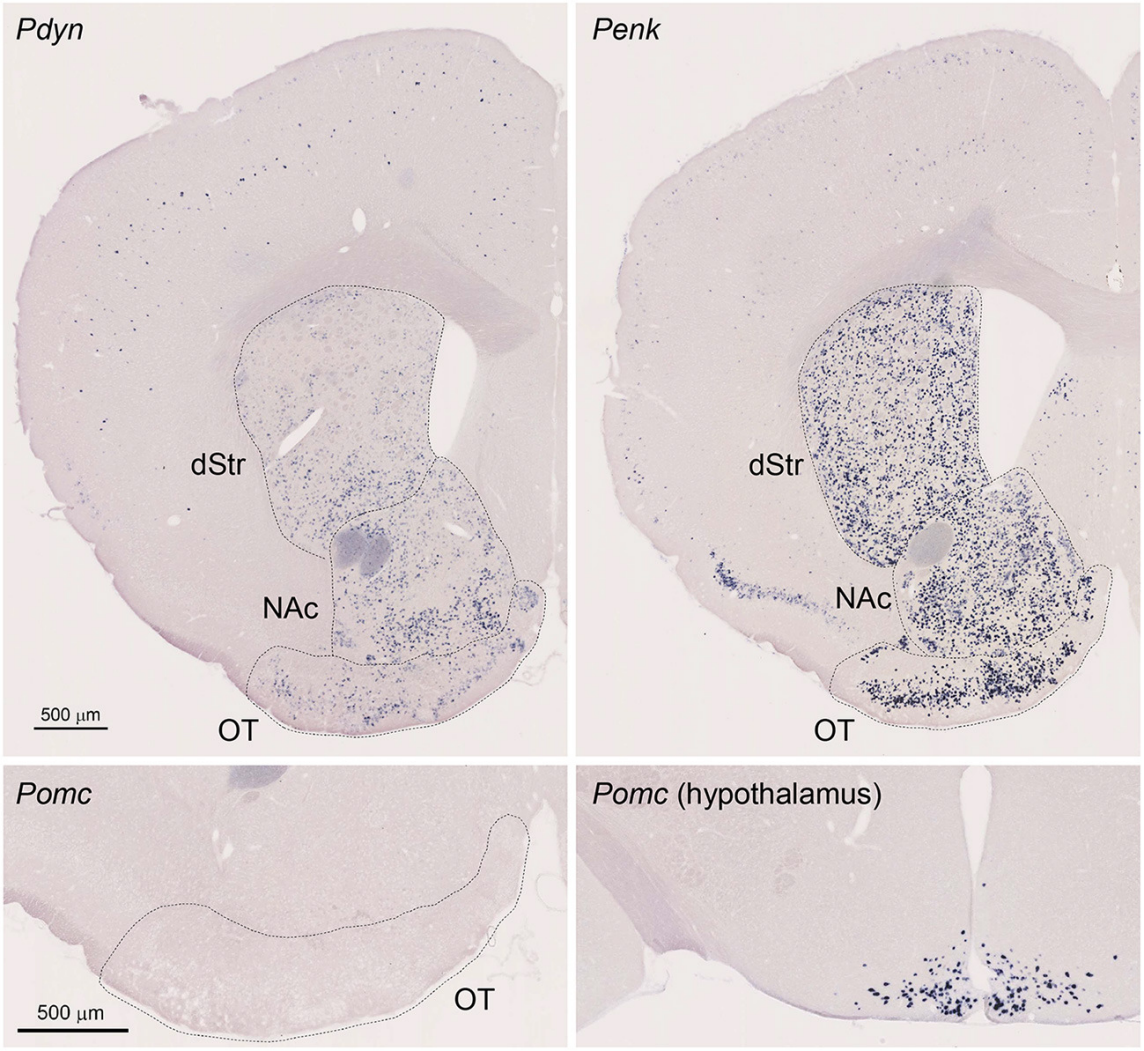
Single Probe *in situ* hybridization (ISH) for Pdyn and Penk in the mouse striatum. Pictures show coronal sections of the anterior OT [approximately at Bregma +1.70 mm of Paxinos and Franklin's the Mouse Brain in Stereotaxic Coordinates (Franklin and Paxinos, 2008)] except for the lower right panel showing the hypothalamus (approximately at Bregma −1.58 mm). Upper left panel; Pdyn, Upper right panel Penk, Lower panels; Pomc. OT, olfactory tubercle; NAc, nucleus accumbens; dStr, dorsal striatum.

**Figure 2:**
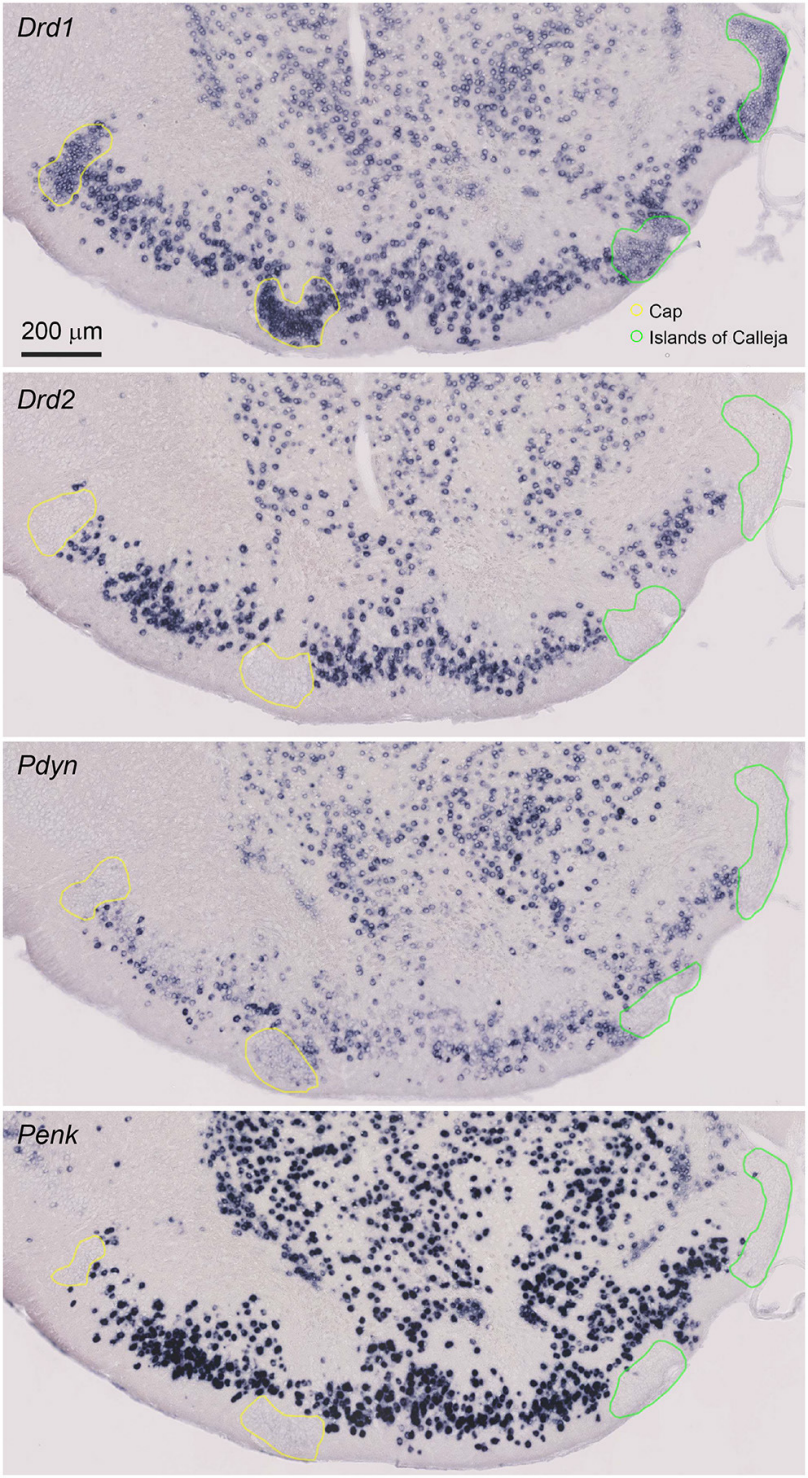
Magnified view of the single probe ISH for Drd1, Drd2, Pdyn, and Penk in the anterior OT. Pictures show coronal sections of the anterior OT (approximately at Bregma +1.70 mm). In the OT, ISH signals of Pdyn and Penk were observed in the DCL. Regions delineated by yellow lines are the Cap regions. Regions delineated by green lines are the ICj. Adjacent sections from one mouse were used for the four pictures. Brain sections in this figure were sampled from a different mouse used in Figure 1.

**Figure 3:**
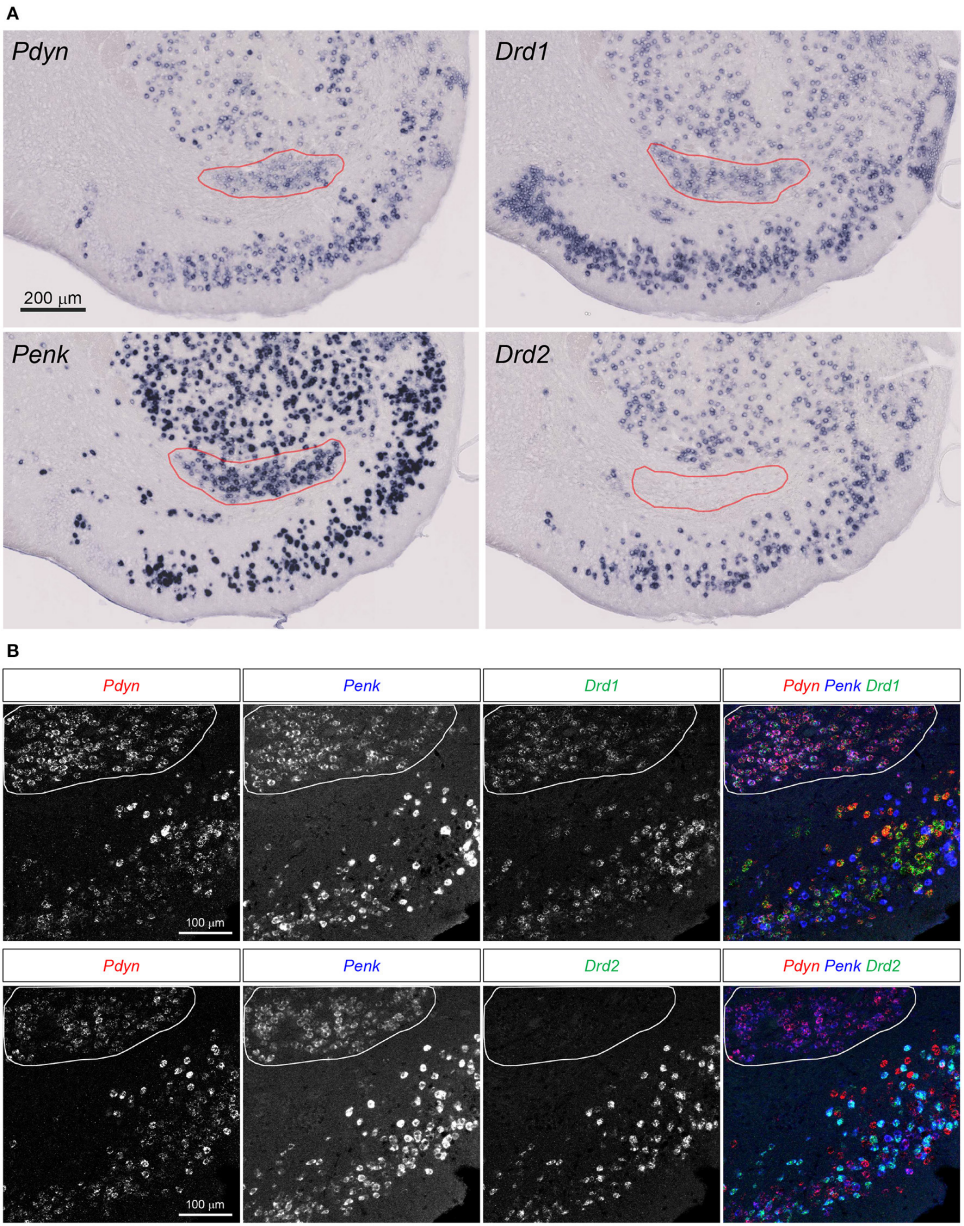
Pdyn-Penk-Drd1 co-expressing cell cluster in the anterior ventral striatum. **(A)** Single probe ISH for Pdyn, Penk, Drd1, and Drd2. The pictures show coronal sections of the anterior OT and NAc (approximately at Bregma +1.94 mm). Regions delineated by red lines are a cluster of Pdyn-Penk-Drd1 co-expressing cells. Drd2 signals were not observed in the cluster. Adjacent sections from one mouse were used for the four pictures. Brain sections in this figure **(A)** were sampled from the same mouse used in Figure 2. **(B)** Triple fluorescence ISH for Pdyn-Penk-Drd1 (Upper panels) and Pdyn-Penk-Drd2 (Lower panels). Regions delineated by white lines are a cluster of Pdyn-Penk-Drd1 co-expressing cells which did not express Drd2.

**Figure 4:**
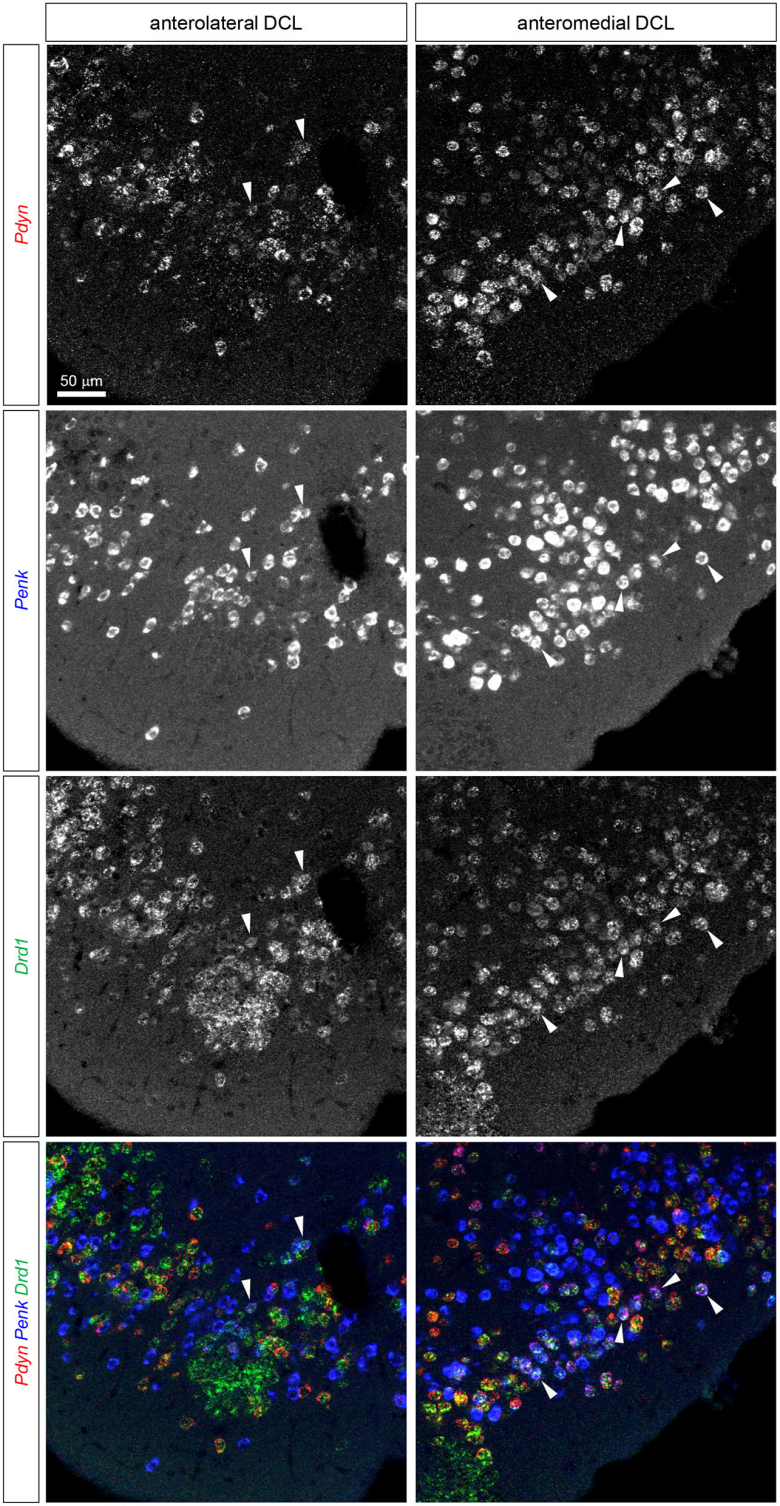
Triple fluorescence ISH for Pdyn-Penk-Drd1 mRNAs in the anteromedial and anterolateral OT DCL. Fluorescence images of Pdyn (red), Penk (blue), and Drd1 (green) mRNA signals in the same region. White arrowheads show the colocalization of Pdyn-Penk-Drd1 mRNAs. Right panels, anteromedial DCL; left panels, anterolateral DCL.

**Figure 5:**
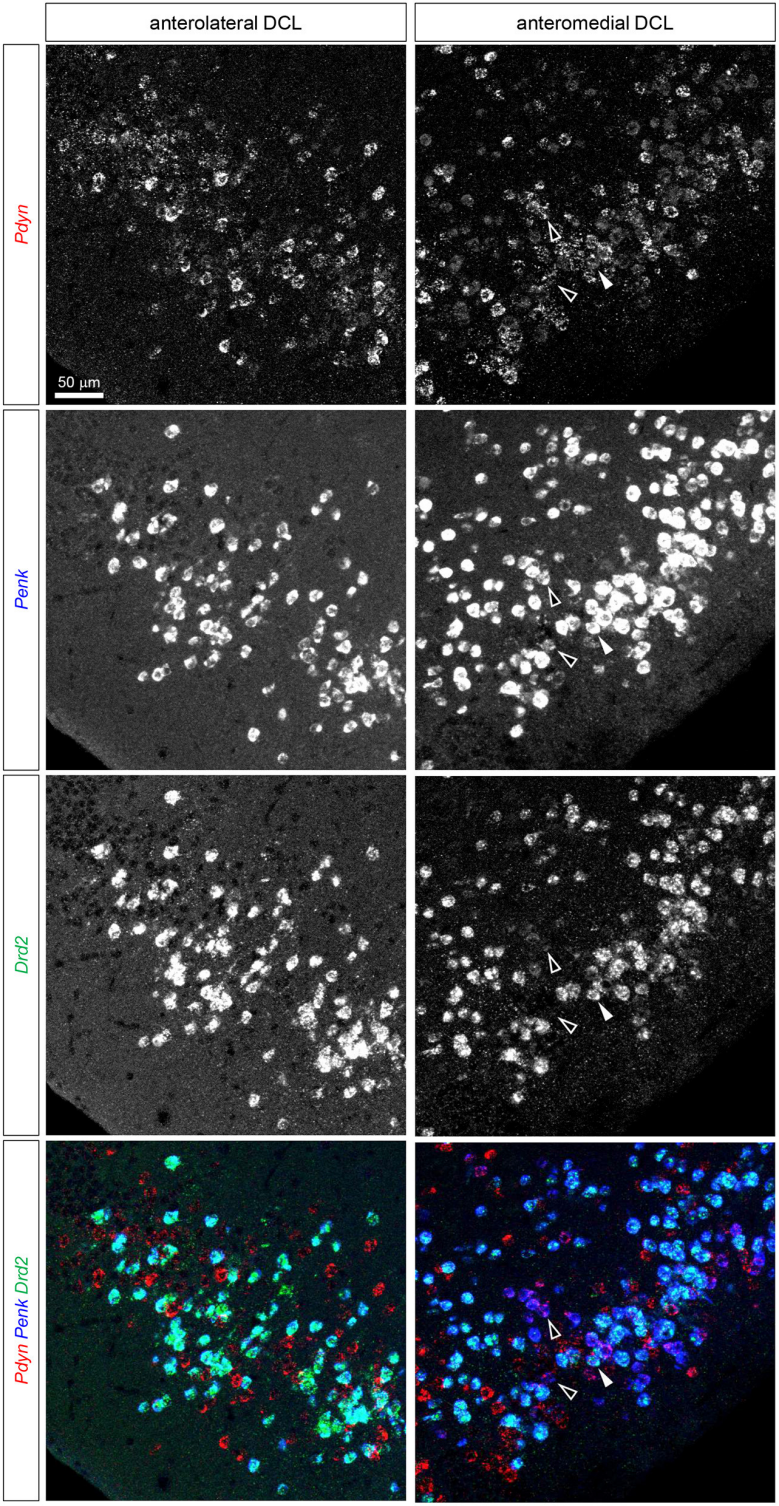
Triple fluorescence ISH for and Pdyn-Penk-Drd2 mRNAs in the anteromedial and anterolateral OT DCL. Fluorescence images of Pdyn (red), Penk (blue), and Drd2 (green) mRNA signals in the same region. A white arrowhead shows the colocalization of the Pdyn-Penk-Drd2 mRNAs. White-outlined arrowheads indicate the colocalization of Pdyn-Penk signals, which did not colocalize with Drd2 signals. Right panels, anteromedial DCL; left panels, anterolateral DCL.

**Figure 6:**
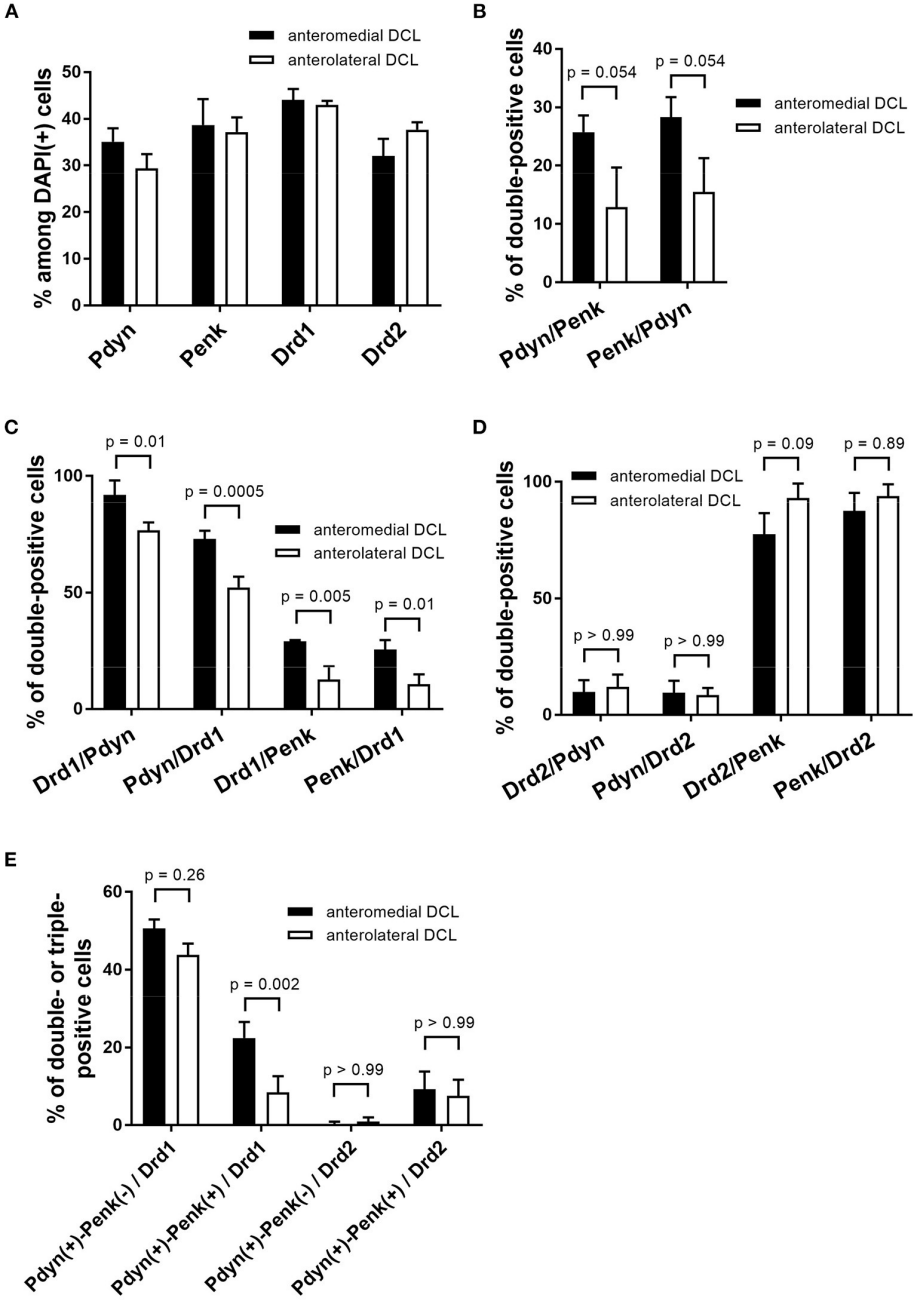
Quantification of Pdyn, Penk, Drd1, and Drd2-expressing cells and their colocalization in the anteromedial and anterolateral OT DCL. **(A)** The percentages were calculated by dividing the number of Pdyn. Penk, Drd1, and Drd2-expressing cells by the number of the DAPI(+) cells in the region of interest. The data of Pdyn and Penk were obtained from Pdyn-Penk-Drd1 co-staining images as shown in Figure 4. **(B–D)**, The percentages of double-positive cells (e.g., Pdyn/Penk shows the percentage of Pdyn-Penk double-positive cells among Penk positive cells in the region of interest). The data in B were obtained from Pdyn-Penk-Drd1 co-staining images as shown in Figure 4. **(E)** The percentage of triple-positive cells (e.g., Pdyn(+)-Penk(-)/Drd1 shows the percentage of the number of Pdyn(+) and Penk(-) Drd1-expressing cells by the number of total Drd1 positive cells in the region of interest). Bars in the graphs represent mean ± SD. *p*-Values were calculated by *post-hoc* Tukey's test after two-way ANOVA. *n* = 3 mice.

**Figure 7:**
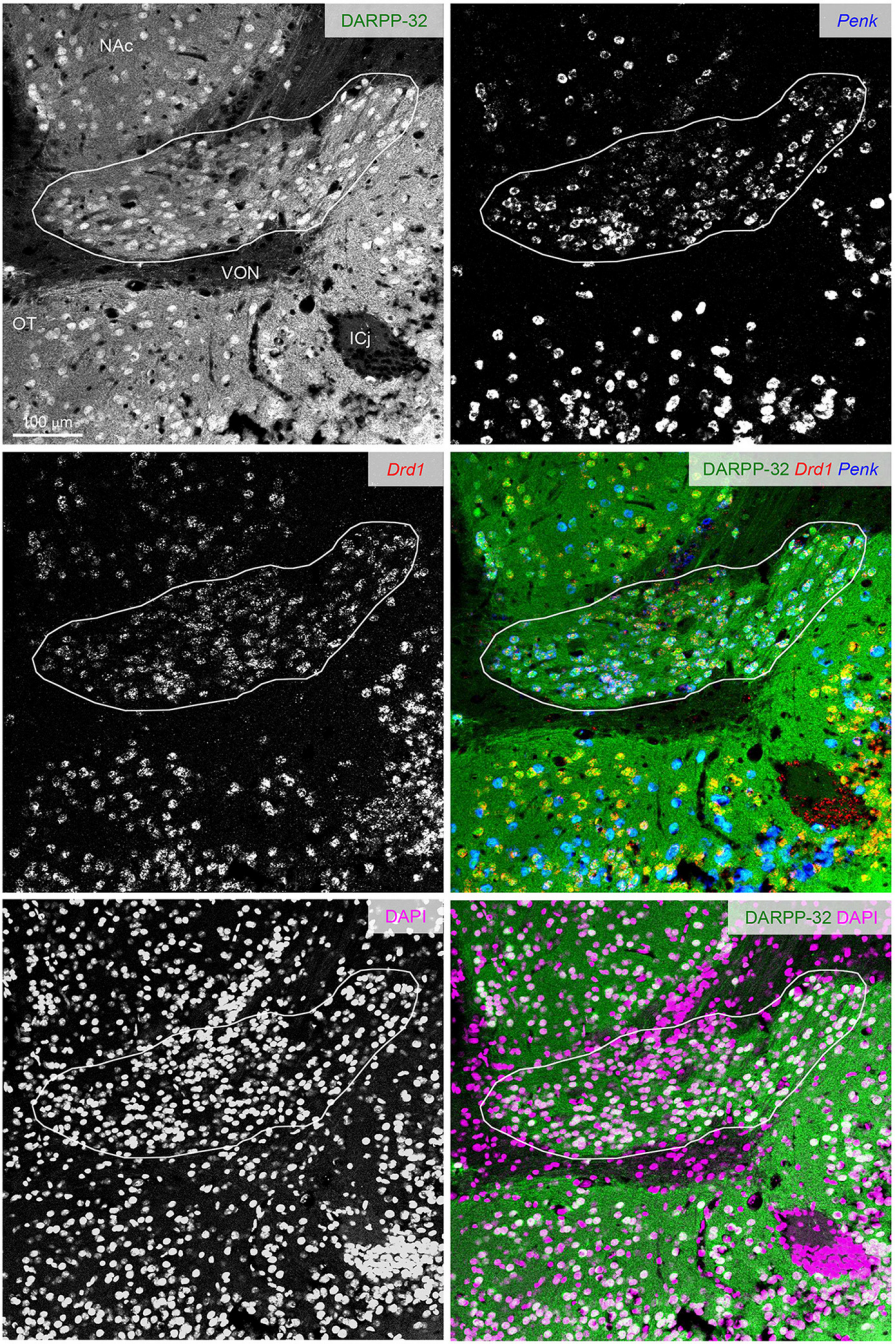
DARPP-32 expression by the putative Pdyn-Penk-Drd1 co-expressing cells in the cell cluster between OT and NAc. Fluorescence images of DARPP-32 immunoreactivity (upper left, green), Penk (upper right, blue), Drd1 (middle left, red) mRNA signals, DAPI (lower left, magenta), and color merged view (middle right; DARPP-32-Drd1-Penk, lower right; DARPP-32-DAPI) in the same region. Regions delineated by white lines are a cluster of putative Pdyn-Penk-Drd1 co-expressing cells. OT, olfactory tubercle; NAc, nucleus accumbens; ICj, Islands of Calleja; VON, ventral olfactory nucleus.

**Figure 8:**
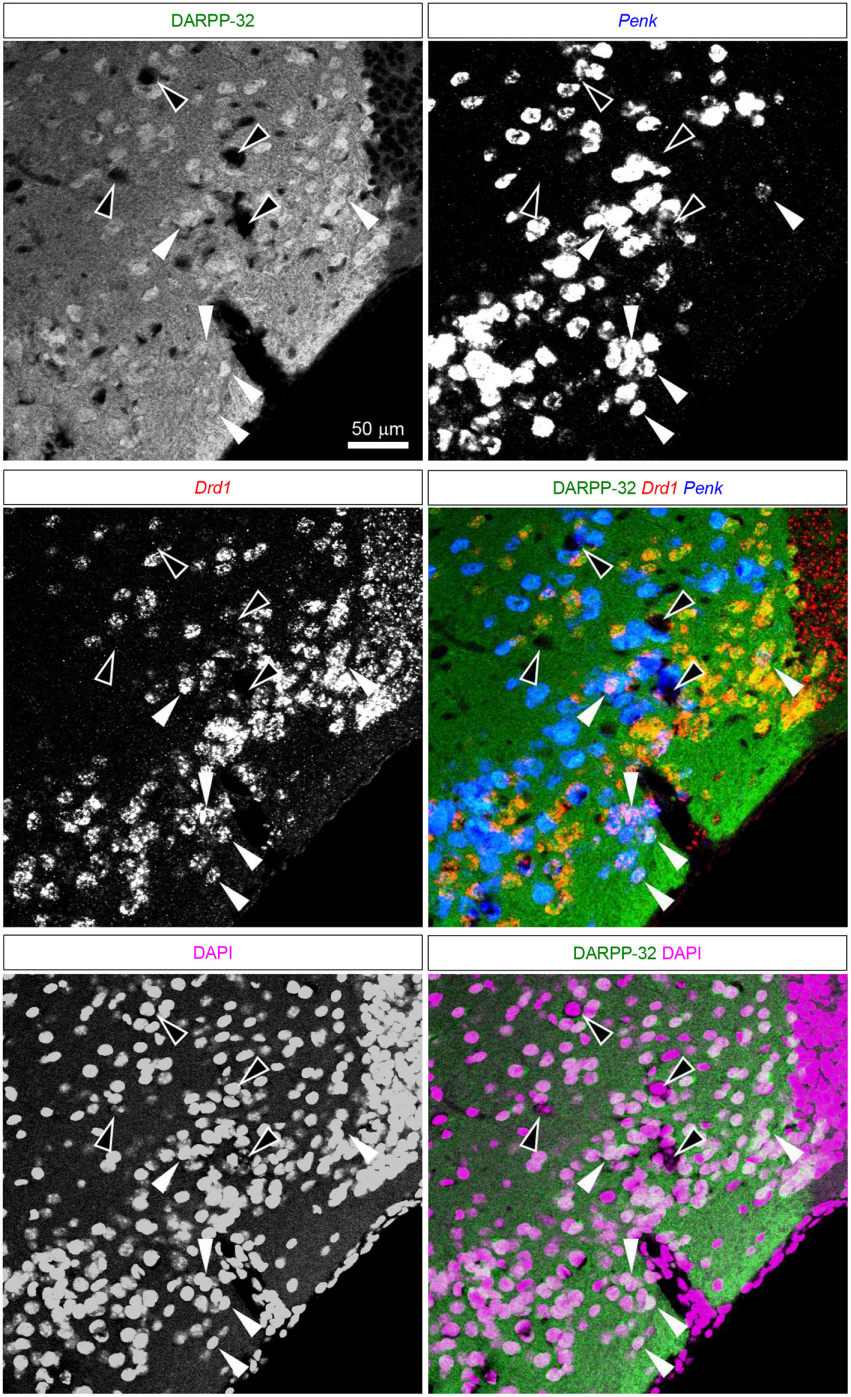
DARPP-32 expression by the putative Pdyn-Penk-Drd1 co-expressing cells in the anteromedial DCL. Fluorescence images of DARPP-32 immunoreactivity (upper left, green), Penk (upper right, blue), Drd1 (middle left, red) mRNA signals, DAPI (lower left, magenta), and color merged view (middle right; DARPP-32-Drd1-Penk, lower right; DARPP-32-DAPI) in the same region. White arrowhead shows the colocalization of the DARPP-32 immunoreactivity and Penk-Drd1 mRNAs. White-outlined arrowheads indicate the colocalization of Penk-Drd1 signals, which did not colocalize with DARPP-32 immunoreactivity.

Male wild-type C57BL/6JJmsSlc mice were obtained from Japan SLC, Inc. The mice were individually housed under a 12/12-h light/dark cycle. Food and water were provided *ad libitum*. We used 14 mice for single-probe ISH, confirming that all the mice had similar ISH signals (Figures 1–3A, Supplementary Figure 1). Of the 14 mice, three were further analyzed for triple fluorescence ISH and quantification of signal colocalization (Figures 3B–8, Supplementary Figures 2, 3).

### Sample Preparation for Histochemistry

Brain samples were obtained from 8 to 12-week-old mice using a previously described method (Murata et al., 2020). Mice were deeply anesthetized by intraperitoneal injection of sodium pentobarbital and transcardially perfused with phosphate-buffered saline (PBS) followed by 4% paraformaldehyde (PFA) in 0.1 M phosphate buffer (PB). The brain was removed from the skull, immersed in 4% PFA in 0.1 M PB overnight, and transferred to 30% sucrose in 0.1 M PB. The brain was then embedded in optimal cutting temperature compound (Sakura Finetek), frozen at −80°C, and sliced into coronal sections with a thickness of 20 μm using a cryotome (CM 3050S, Leica). The brain sections were rinsed in 0.1 M PB, mounted on glass slides (CREST coat, Matsunami) using a paintbrush, dried overnight, and stored in a freezer until histochemical staining.

### RNA Probe Preparation for *in situ* Hybridization

Mouse Pdyn, Penk, and proopiomelanocortin (Pomc) cDNAs were subcloned by conventional PCR using the following primers in accordance with the Allen Brain Atlas (http://mouse.brain-map.org/, Pdyn, Entrez ID 18610; Penk, Entrez ID 18385; Pomc, Entrez ID 18739): Pdyn, 5'-AGGAAAAGTTCAGGGGTCTCTC−3' – 5'-TCTCACAGTTCCCATGCAATAC−3'; Penk, 5'-TTCCTGAGGCTTTGCACC-3' – 5'-TCACTGCTGGAAAAGGGC-3'; andPomc,5'-CGACGGAAGAGAAAAGAGGTTA-3' – 5'-CTTGGAATGAGAAGACCCCTG-3'. We used a commercial mouse brain cDNA library (MD-01, Genostaff) as amplification templates. The PCR products were subcloned into pGEM-T Easy plasmids (Promega) for *in vitro* transcription of RNA probes. Plasmid templates for Drd1 and Drd2 probes were provided by Dr. Kazuto Kobayashi (Sano et al., 2003). We prepared digoxigenin (DIG)-, fluorescein (FLU)-, and biotin (BIO)-labeled RNA probes using an *in vitro* transcription kit (Roche) according to the manufacturer's protocol. We prepared both antisense and sense probes for opioid precursors and dopamine receptors, and confirmed that ISH using sense probes yielded no detectable signals (data not shown). All data presented in this report were obtained by ISH using antisense probes.

### ISH Using a Single Probe

ISH using a single probe for Pdyn, Penk, Pomc, Drd1, and Drd2 (Figures 1–3A, Supplementary Figure 1) was performed using a previously described method (Murata et al., 2020). Brain sections were fixed in 4% PFA for 20 min, digested with proteinase K (10 μg/mL) for 30 min, and post-fixed in 4% PFA for 15 min. After pre-hybridization, the sections were incubated overnight at 65°C with DIG-labeled RNA probes. After stringent washing, the sections were blocked with 10% normal sheep serum, 1% bovine serum albumin, and 0.1% Triton X-100 in PBS. Subsequently, the sections were incubated overnight at 4°C with an alkaline phosphatase-conjugated anti-DIG antibody (1:1,000; Roche). The sections were washed in Tris-NaCl-Tween 20 (TNT) buffer (0.1 M Tris-HCl; pH 7.5; 0.15 M NaCl; 0.1% Tween 20), followed by alkaline phosphatase buffer (100 mM NaCl; 100 mM Tris-HCl; pH 9.5; 50 mM MgCl_2_; 0.1% Tween 20; 5 mM levamisole). The sections were treated overnight with 5-bromo-4-chloro-3-indolyl-phosphate/nitro blue tetrazolium (BCIP/NBT) color development substrate mixture (Promega) at room temperature in a dark room for color development. They were then rinsed in PBS and mounted in Mount-Quick Aqueous (Daido Sangyo).

### Triple Fluorescence ISH Using DIG-, FLU-, and BIO-Labeled Probes

Triple fluorescence ISH using DIG-, FLU-, and BIO-labeled probes (Figures 3B–8, Supplementary Figures 2, 3) was performed using a modification of a previously described method (Konno et al., 2014; Murata et al., 2020). Hybridization and washing were performed as described above, except that the DIG, FLU, and BIO probes were mixed and used for hybridization. After blocking in 1% blocking buffer (11096176001, Roche) for 1 h, the DIG-, FLU-, and BIO-labeled probes were detected by fluorescence. For detection of FLU-labeled probes, the sections were incubated with an anti-FLU antibody conjugated with horseradish peroxidase (1:500; Perkin-Elmer) for 1 h at room temperature. After three 10-min washes in TNT, the sections were treated with diluted (1:100) Tyramide Signal Amplification (TSA)-Plus FITC reagents for 10 min according to the manufacturer's instructions (Perkin-Elmer), and the FLU signals were converted to FITC signals. The sections were then washed in TNT for 10 min three times, and incubated in 0.02 N HCl-TNT for 30 min at room temperature to inactivate peroxidase activity associated with the detection of the previous RNA probes. To detect DIG-labeled probes, the sections were incubated with an anti-DIG antibody conjugated with horseradish peroxidase (1:500; Perkin-Elmer) for 1 h at room temperature. After three 10-min washes in TNT, the sections were treated with diluted (1:100) TSA-Plus Cy3 reagents for 10 min according to the manufacturer's instructions (Perkin-Elmer), and the DIG signals were converted to Cy3 signals. The sections were then washed in TNT for 10 min three times, and again incubated in 0.02 N HCl-TNT for 30 min at room temperature. To detect BIO-labeled probes, the sections were incubated with an anti-BIO antibody conjugated with horseradish peroxidase (1:500; Funakoshi) for 1 h at room temperature. After three 10-min washes in TNT, the sections were treated with diluted (1:100) TSA-Plus Cy5 reagents for 10 min according to the manufacturer's instructions (Perkin-Elmer), and the BIO signals were converted to Cy5 signals. The sections were then counterstained with DAPI diluted in PBS (1 μg/mL) for 5 min. After washing with PBS, the sections were mounted using Prolong Glass Antifade Mountant (Thermo Fisher Scientific).

### Triple Fluorescence Labeling of DARPP-32 Immunoreactivity With ISH Using DIG- and FLU-Labeled Probes

In Figures 7, 8, DIG-labeled Drd1 and FLU-labeled Penk probes were labeled by Cy3 and Cy5 fluorescence signals as described above, respectively. To detect DARPP-32 immunoreactivity, the sections were then incubated with an anti-DARPP-32 antibody (1:500, Abcam ab40801) overnight at 4°C, followed by TNT wash and secondary antibody incubation (Alexa 488 conjugated-donkey anti-rabbit IgG antibody. 1:500; Jackson Immunoresearch Laboratories Inc. 711-545-152) for 1 h at room temperature. The sections were counterstained with DAPI, washed with PBS, and mounted using Prolong Glass Antifade Mountant.

### Image Acquisition and Analysis

The BCIP/NBT color-developed samples were examined using a bright-field virtual slide system (NanoZoomer, Hamamatsu Photonics) (Figures 1–3A, Supplementary Figure 1). A confocal fluorescent laser microscope (FV2000, Olympus) was used to obtain fluorescence images, as shown in Figures 3B–8, Supplementary Figures 2, 3. ImageJ software was used to perform cell counts and signal colocalization (Figures 3B–8). We sought to avoid bleaching the fluorescent signals by restricting the section exposure to light as much as possible. Three to four coronal sections per mouse were used for obtaining confocal images of Pdyn-Penk-Drd1 and Pdyn-Penk-Drd2 fluorescence labeling. One coronal section was then chosen for cell quantification in Figure 6. The number of DAPI(+) cells examined for their expression and the colocalization of Pdyn, Penk, Drd1, and Drd2 are described in Supplementary Table 2.

### Statistics

The normality of data was first tested using the Shapiro-Wilk test in JASP ver. 0.16. The data are presented as the mean ± SD. Statistical differences were analyzed by two-way ANOVA (cell-types x anteromedial/anterolateral DCL) with *post-hoc* Tukey's test using GraphPad Prism 7 (*n* = 3 mice, Figure 6).

## Results

### Expression of Pdyn and Penk mRNAs in the Mouse Anterior OT

We first confirmed that Pdyn and Penk mRNAs were expressed in the anterior OT, dorsal striatum, and NAc using single-probe ISH in mice (Figure 1). Penk mRNA signals were also observed in the piriform cortex, with a tendency toward high expression in the dorsal part. Pdyn mRNA was not detected in the piriform cortex. ISH staining for Pomc mRNA, a precursor gene of β-endorphin, showed no significant signals in the OT, whereas it successfully labeled neurons in the arcuate nucleus of the hypothalamus (Gee et al., 1983; Lewis et al., 1986).

The cytoarchitecture of the OT can be defined by the expression of the Drd1 and Drd2 mRNAs. We compared the Drd1 and Drd2 ISH signals with the Pdyn and Penk signals in the neighboring OT coronal sections (Figure 2, Supplementary Table 1). The OT is a three-layered structure with several cell clusters. The DCL (layer II) contains the MSNs, where Drd1- and Drd2-expressing neurons are intermingled. Both Pdyn- and Penk-expressing neurons were observed in the DCL, suggesting that MSNs express Pdyn and/or Penk mRNA. The Cap regions are cell-clustered regions in the lateral part of the OT that contain dwarf cells (Figure 2, yellow lines), which express Drd1 but not Drd2. A weak Pdyn signal was detected in the Cap region, but Penk signals were not detected in the Cap region. The ICj is another cell-clustered region distributed in the medial part of the OT (Figure 2, green lines). The ICj contains granule cells that weakly express Drd1 with no detectable Drd2 mRNA. Pdyn and Penk signals were not detected in the ICj.

Curran and Watson reported Pdyn-, Penk-, and Drd1-coexpressing cell clusters in the rat ventral striatum (Curran and Watson, 1995). We confirmed that the similar cell clusters expressing the three genes in the mouse ventral striatum (Figure 3A, Supplementary Figure 1). Drd2 mRNA signals were not detected in the cell clusters. They were distributed between the anterior NAc and OT, and separated into the medial and lateral parts in the posterior sections (Figure 3A, Supplementary Figure 1, red lines).

### Examination of the Co-expression of Pdyn, Penk, Drd1, and Drd2 mRNAs in the DCL of the Anteromedial and Anterolateral OT by Triple Fluorescence ISH

Here, we employed triple fluorescence ISH for Pdyn-Penk-Drd1 and Pdyn-Penk-Drd2 to perform cell typing of Pdyn and Penk expression by Drd1- and Drd2-expressing cells as well as to address whether Pdyn-Penk-Drd1 cells are distributed in the DCL of the OT. Triple fluorescence ISH demonstrated that the cell cluster between the NAc and OT co-expressed Pdyn-Penk-Drd1 but not Drd2 (Figure 3B). This observation also confirmed successful multiple labeling and separation of the mRNAs by fluorescence ISH (Figures 4, 5, Supplementary Figures 2, 3).

The DCL of the OT comprises Drd1-, Drd2-, Pdyn-, and Penk-expressing cells (Figures 2, 3). Previous studies in the NAc revealed that Pdyn is mainly expressed by Drd1-expressing cells, whereas Penk is primarily expressed by Drd2-expressing cells (Curran and Watson, 1995). We analyzed triple fluorescence ISH for Pdyn-Penk-Drd1 and Pdyn-Penk-Drd2 to determine whether the DCL of the OT showed a similar co-expression to the NAc and whether Pdyn-Penk-Drd1 co-expressing neurons were also distributed in the DCL (Figures 4–6, Supplementary Figures 2, 3). We hereafter separated the DCL into anteromedial and anterolateral domains. We used the Cap region and ICj as the physical boundary of the anteromedial and anterolateral OT. The DCL surrounded by the Cap regions in the anterior OT was referred to as anterolateral. The DCL surrounded by the ICj that protrudes to the ventromedial brain surface was referred to as anteromedial. We used the coronal sections that include superficially located ICj (approximately bregma +1.18-1.98 mm, Franklin and Paxinos, 2008) as the anterior OT.

We observed expressions of the Drd1, Drd2, Pdyn, and Penk mRNAs in the DCL of the anteromedial and anterolateral OT (Figures 4, 5, Supplementary Figures 2, 3). The percentages of single mRNA expression for Pdyn, Penk, Drd1, and Drd2 among DAPI(+) cells in the anteromedial and anterolateral DCL were Pdyn: 35.1% and 29.3%, Penk: 38.7% and 37.2%, Drd1: 44.1% and 43.0%, and Drd2: 32.0% and 37.7%, respectively (Figure 6A, Supplementary Table 2).

We then examined the co-expression of Pdyn-Penk, Pdyn-Drd1, Penk-Drd1, Pdyn-Drd2, and Penk-Drd2 mRNAs and compared the percentages of each combination of the anteromedial and anterolateral DCL (Figures 6B–D). To summarize, most MSNs in the OT DCL showed colocalization of Pdyn-Drd1 or Penk-Drd2 mRNAs, which is consistent with the MSNs in the NAc. Colocalization of Pdyn and Penk mRNAs occurred as follows: in the anteromedial DCL, Pdyn/Penk, 25.7%; and Penk/Pdyn, 28.3%. In the anterolateral DCL, 12.9% were Pdyn/Penk and 15.5% were Penk/Pdyn. Although the differences between the two regions were not statistically significant, the percentages of the Pdyn and Penk co-expression were higher in the anteromedial DCL than in the anterolateral DCL (p = 0.054 for both Pdyn/Penk and Penk/Pdyn) (Figure 6B). Most Pdyn(+) cells expressed Drd1 (anteromedial DCL, 91.9%; anterolateral DCL, 76.7%), and more than half of the Drd1(+) cells expressed Pdyn (anteromedial DCL, 73.0%; anterolateral DCL, 52.3%). The percentages of Drd1/Pdyn and Pdyn/Drd1 in the anteromedial DCL were significantly higher than those in the anterolateral DCL (Figure 6C). Compared to the Pdyn, Drd1-expressing neurons showed smaller percentages of colocalization with Penk in both anteromedial DCL (Drd1/Penk; 29.1%, and Penk/Drd1; 25.7%) and anterolateral DCL (Drd1/Penk; 12.6%, and Penk/Drd1; 10.6%) (Figure 6C). The percentages of Drd1-Penk colocalization was also higher in the anteromedial DCL than in the anterolateral DCL (Figure 6C). A majority of Penk(+) cells expressed Drd2 (anteromedial DCL, 77.6%; anterolateral DCL, 93.1%), and most Drd2(+) cells expressed Penk (anteromedial DCL, 87.5%; anterolateral DCL, 93.9%) (Figure 6D). In contrast, a smaller population of Pdyn-Drd2 colocalization was observed in both the anteromedial DCL (Drd2/Pdyn: 9.8%, and Pdyn/Drd2: 9.5%) and anterolateral DCL (Drd2/Pdyn, 12.0%; and Pdyn/Drd2, 8.5%) (Figure 6D).

We then examined the existence of Pdyn-Penk-Drd1 triple-positive cells in the anteromedial and anterolateral DCL. We observed that 22.4% of Drd1-expressing cells in the anteromedial DCL co-expressed both Pdyn and Penk, which was significantly higher than the percentage in the anterolateral DCL (8.5%) (Figure 4, white arrowheads, and Figure 6E). We also found that 9.6% and 7.6% of the Drd2-expressing cells co-expressed both Pdyn and Penk in the anteromedial and anterolateral DCL, respectively (Figure 5, white arrowhead, and Figure 6E).

### Expression of DARPP-32 by the Pdyn-Penk-Drd1 Co-expressing Cells in the Anterior OT

The ventral pallidum anteriorly protrudes into the OT deep layer (multiform layer), where pallidal cells are distributed (Heimer et al., 1987; Root et al., 2015). This raises the possibility of the Pdyn-Penk-Drd1 co-expressing cells are pallidal cells in the anterior OT (Figures 3, 4, Supplementary Figure 1). Here, we examined the DARPP-32 expression of the Pdyn-Penk-Drd1 cells in the cell cluster between anterior OT and NAc (Figure 7) and anteromedial DCL (Figure 8). DARPP-32 immunoreactivity is present in somata and dendrites of striatal cells but not in somata of pallidal cells (Ouimet et al., 1984). Because most Penk-Drd1 co-expressing cells in the DCL expressed Pdyn (85.6 ± 6.9%, Supplementary Table 2), we regarded Penk-Drd1 cells as putative Pdyn-Penk-Drd1 cells.

The majority of the putative Pdyn-Penk-Drd1 neurons expressed DARPP-32 in the cell cluster between anterior OT and NAc (93.7 ± 4.5%, *n* = 3 mice) and anteromedial DCL (83.7 ± 2.1%, *n* = 3 mice). None of the DARPP-32 immunonegative cells in the multiform layer of the anteromedial OT showed Penk or Drd1 mRNA signal (Figure 8 white-outlined arrowheads, 33, 40, and 69 cells from three mice). This neurochemical feature of DARPP-32 immunopositivity supports the idea that the Pdyn-Penk-Drd1 cells in the anterior OT are striatal cells and can be distinguished from DARPP-32 negative putative pallidal cells. We note that the DARPP-32 immunonegative small region dorsally adjacent to the OT (Figure 7) is the ventral olfactory nucleus, where GABAergic neurons project their axons to the lateral hypothalamus (Murata et al., 2019b).

## Discussion

In the current study, we performed multiple ISH to reveal the cellular profile of Pdyn and Penk mRNA expression in Drd1- and Drd2-expressing cells in the anterior OT of mice. MSNs in the DCL express Pdyn, Penk, or both. Dwarf cells in the Cap region showed weak Pdyn and no Penk signals. In the granule cells of the ICj, the Pdyn and Penk signal intensities were below detection sensitivity. In the MSNs of the DCL, Pdyn was mainly expressed by Drd1-expressing neurons, whereas Penk was expressed primarily by Drd2-expressing neurons, which is consistent with previous reports in the NAc (Curran and Watson, 1995). We confirmed a cell cluster of Pdyn-Penk-Drd1 triple-positive cells in the ventral striatum of mice, as previously reported in rats (Curran and Watson, 1995). We found that the Pdyn-Penk-Drd1 triple-positive cells in the DCL of the anterior OT and the percentage of Pdyn-Penk co-expressing cells among Drd1-expressing cells was higher in the anteromedial DCL than in the anterolateral DCL. The Pdyn-Penk-Drd1 cells in the anteromedial DCL expressed DARPP-32, which suggests their striatal feature in terms of molecular expression. We also found a small population of Drd2-expressing cells that co-expressed Pdyn and Penk in the DCL of the anterior OT.

In this study, we used an exploratory approach to address the mechanisms of the distinct roles of Drd1- and Drd2-expressing cells in anteromedial and anterolateral OT by examining Pdyn and Penk expression. A significant difference between Drd1- and Drd2-expressing cells in the DCL is their expression of Pdyn and Penk, respectively. A remarkable difference between the anteromedial and anterolateral DCL was the larger percentage of Pdyn-Penk co-expressing Drd1 cells in the anteromedial DCL (Figure 4, white arrowheads, and Figure 6E). This observation raises a possible new subtype of Pdyn-Penk-Drd1 co-expressing cells in anteromedial DCL. The Pdyn-Penk co-expressing Drd1 cells in the ventral striatum were initially reported by Curran and Watson using rats (Curran and Watson, 1995), which showed a similar distribution to the Pdyn-Penk-Drd1 co-expressing cell cluster in the current mouse study (Figure 3A, Supplementary Figure 1). Distribution of the Pdyn-Penk-Drd1 co-expressing cell clusters was coincided with the lateral stripe of the striatum (LSS) and LSS-associated cell clusters in rats (Zhou et al., 2004). Zhou, Furuta, and Kaneko revealed that neurons in the LSS and LSS-associated cell clusters express neurokinin B and project their axons to the interstitial nucleus of the posterior limb of the anterior commissure and the substantia innominata. Currently, the neural connectivity of the Pdyn-Penk-Drd1 co-expressing cells in the OT DCL is unknown. Axonal projection of the Pdyn-Penk-Drd1 cells in the DCL will distinguish whether they are similar to LSS and LSS-associated neurons or MSNs in the DCL which project to the ventral pallidum (VP) (Heimer et al., 1987; Zhang et al., 2017b). Input pathways to the Pdyn-Penk-Drd1 cells in the DCL will also be essential to characterize their neuronal profiles. Although DARPP-32 expression supports their striatal features, it remains to be examined whether they receive dopaminergic innervation from the ventral tegmental area (Ikemoto, 2007). It also remains to be confirmed whether the Pdyn-Penk-Drd1 cells in the DCL receive synaptic inputs from the olfactory bulb and other olfactory cortical areas (Zhang et al., 2017b).

Our histochemical examination revealed the molecular profiles of dopamine receptors and opioid precursor peptides of MSNs in the anteromedial and anterolateral OT. Drd1-expressing MSNs in the anteromedial and anterolateral OT express Pdyn, which are involved in odor-induced attractive and aversive behaviors, respectively. Drd2-expressing MSNs in the anteromedial OT express Penk, which are involved in odor-induced aversive behavior (Murata et al., 2015). These data will help further experiments of optogenetics and behavioral pharmacology examining whether and how dynorphins and enkephalins released by the OT MSNs influence odor-induced attractive and aversive behaviors. The Pdyn-Penk co-expressing Drd1 cells can transmit to both kappa (KOR) and delta (DOR) opioid receptor-expressing neurons (Mansour et al., 1988, 1994). Co-transmission to KOR and DOR neurons from the anteromedial DCL of the OT might also underlie the odor-induced attractive behavior (Murata et al., 2015). Zhou et al. showed that both preprodynorphin- and preproenkephalin peptide-expressing cells in the OT project their axons to the ventrolateral part of the VP (Zhou et al., 2003). Al-Hasani et al. have demonstrated *in vivo* detection of optically-evoked endogenous opioid peptide release by neurons in the NAc (Al-Hasani et al., 2018), which supports the idea that neural activation of the OT MSNs will lead to release of dynorphins and enkephalins in their target regions. Microinjection of a mu-opioid receptor agonist into the posterior part of the VP elicited enhanced hedonic reactions to the oral infusion of sucrose solution, whereas the same microinjection into the anterior and central parts of the VP suppressed the hedonic reaction (Smith and Berridge, 2007). A recent study revealed that the neural pathway of the posterior olfactory bulb to the OT is related to the attraction to pleasant odorants (Midroit et al., 2021). Future studies should address whether and how Pdyn- and Penk-expressing cells in the OT and their axonal projections to the VP are related to the pleasantness of olfaction.

## Data Availability Statement

The original contributions presented in the study are included in the article/Supplementary Material, further inquiries can be directed to the corresponding authors.

## Ethics Statement

The animal study was reviewed and approved by the Experimental Animal Research Committee of the University of Fukui.

## Author Contributions

AM and KM designed the study, performed the experiments, and wrote the manuscript. KK, SF, and YF contributed tools and reagents and assisted in the revision of the manuscript. All authors reviewed and approved the final manuscript.

## Funding

KM was supported by JSPS KAKENHI Grant Numbers 17KK0190, 18H05005, 21H05817, and 21K06440; the Lotte Foundation; the Takeda Science Foundation; and Research Grants from the University of Fukui (FY 2021). SF was supported by JSPS KAKENHI (Grant Numbers 21H03086 and 21K19559); the Japan Agency for Medical Research and Development (AMED, number 21452955); and a Health Labor Sciences Research Grant [R3-Nantitou (nan)-Ippan-21FC1013]. YF was supported by JSPS KAKENHI (Grant Numbers 19H03323 and 20H05058).

## Conflict of Interest

KM received research funding from the LOTTE Foundation. The remaining authors declare that the research was conducted in the absence of any commercial or financial relationships that could be construed as a potential conflict of interest.

## Publisher's Note

All claims expressed in this article are solely those of the authors and do not necessarily represent those of their affiliated organizations, or those of the publisher, the editors and the reviewers. Any product that may be evaluated in this article, or claim that may be made by its manufacturer, is not guaranteed or endorsed by the publisher.
