# Supplementary material for: Cellular Profiles of Prodynorphin and Preproenkephalin mRNA-Expressing Neurons in the Anterior Olfactory Tubercle of Mice
Source: Front Neural Circuits. 2022 Jul 22;16:908964. doi: 10.3389/fncir.2022.908964 (PMC9352893; doi:10.3389/fncir.2022.908964)
Supplement: Supplementary file 1 [file Presentation_1.PDF]

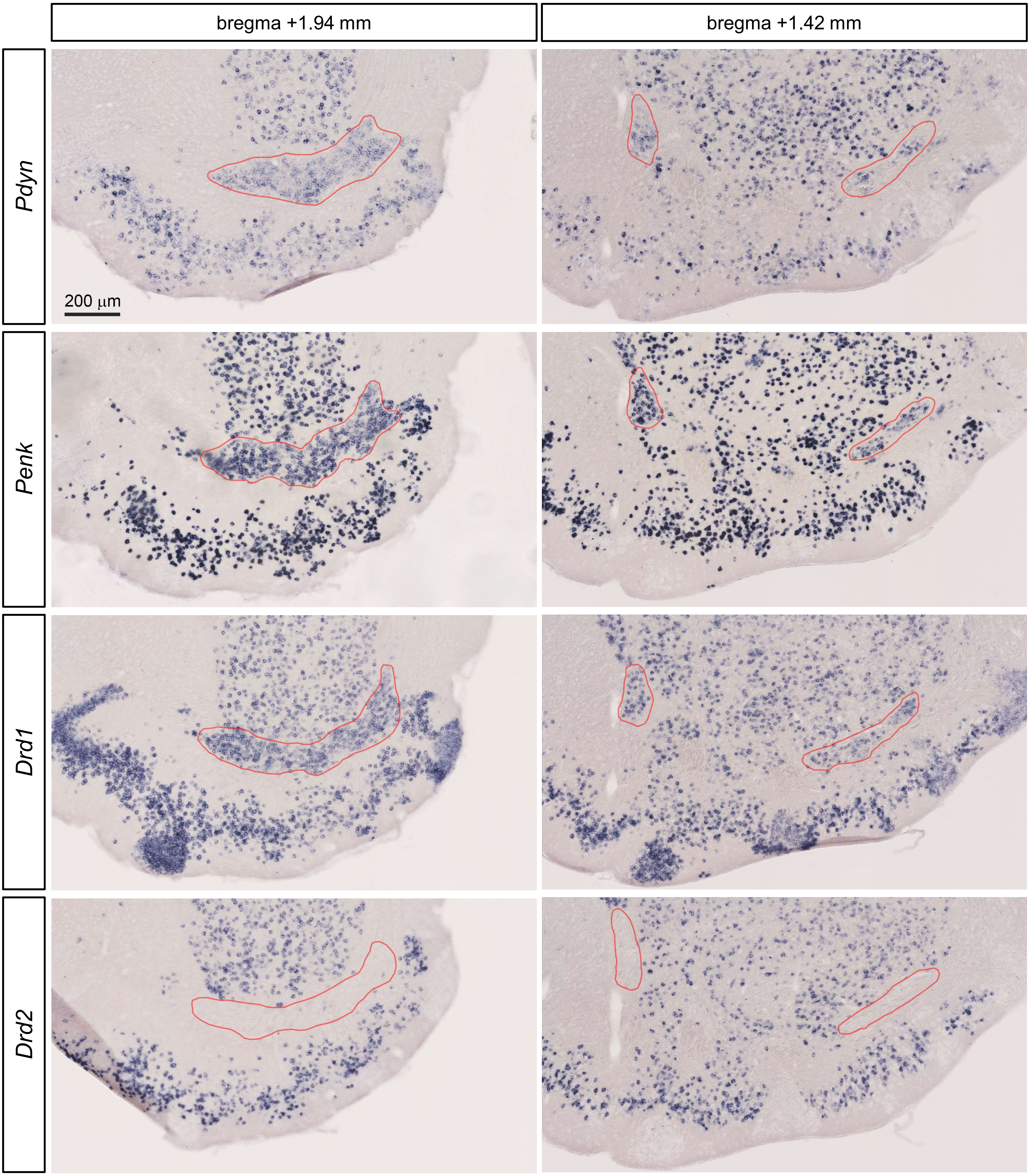

Supplementary Figure 1. Distribution of Pdyn-Penk-Drd1 co-expressing cell cluster in the anterior ventral striatum. Images are single probe ISH for Pdyn, Penk, Drd1, and Drd2. The pictures show coronal sections of the anterior OT and NAc (approximately at Bregma +1.94 mm left column and +1.42 mm right column). Regions delineated by red lines are clusters of Pdyn-Penk-Drd1 co-expressing cells. Drd2 signals were not observed in the cluster.

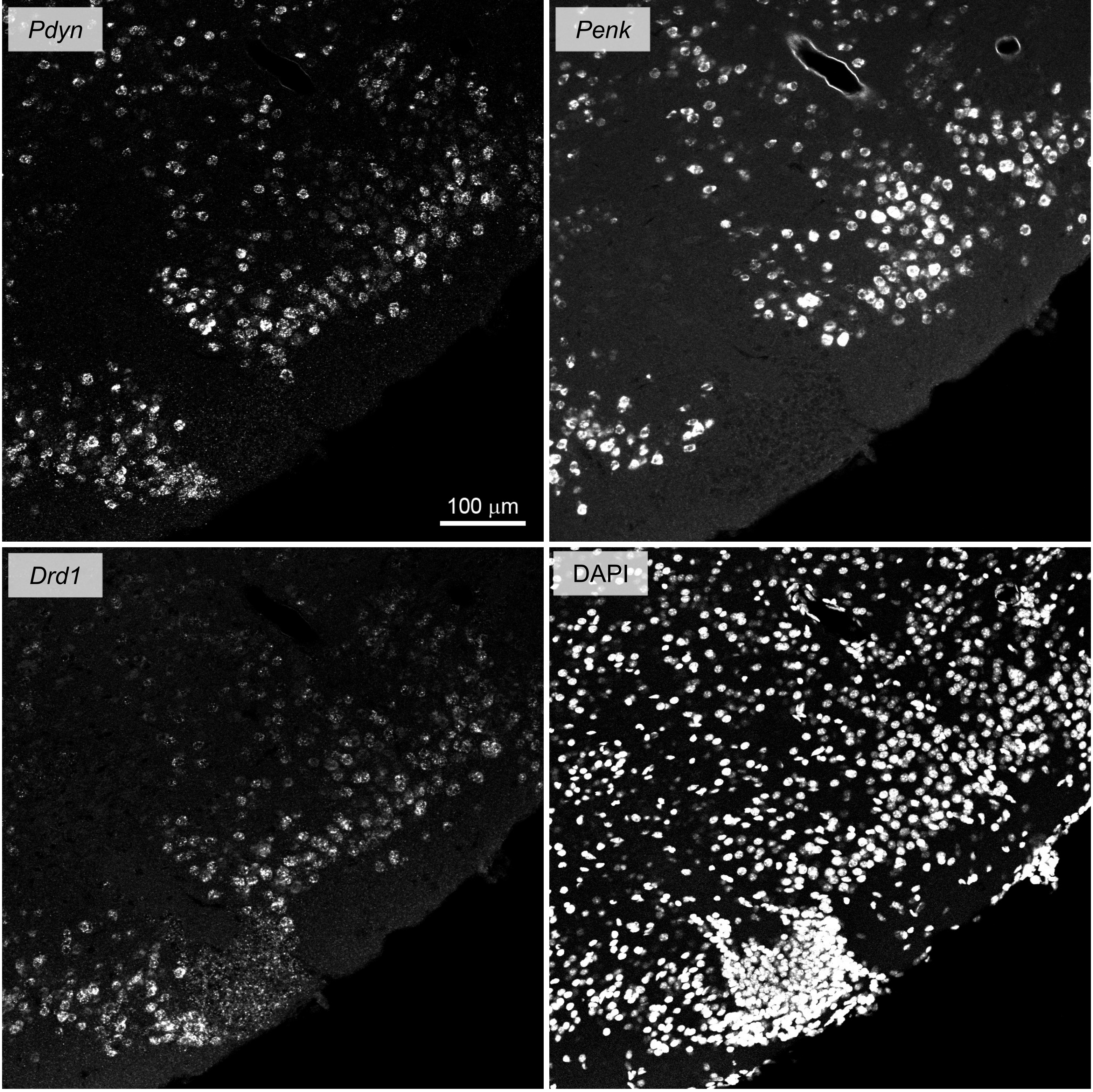

Supplementary Figure 2. DAPI(+) cells in the anteromedial DCL with triple fluorescence ISH signals of Pdyn-Penk-Drd1. Fig. 4 right panels were obtained by cropping these pictures.

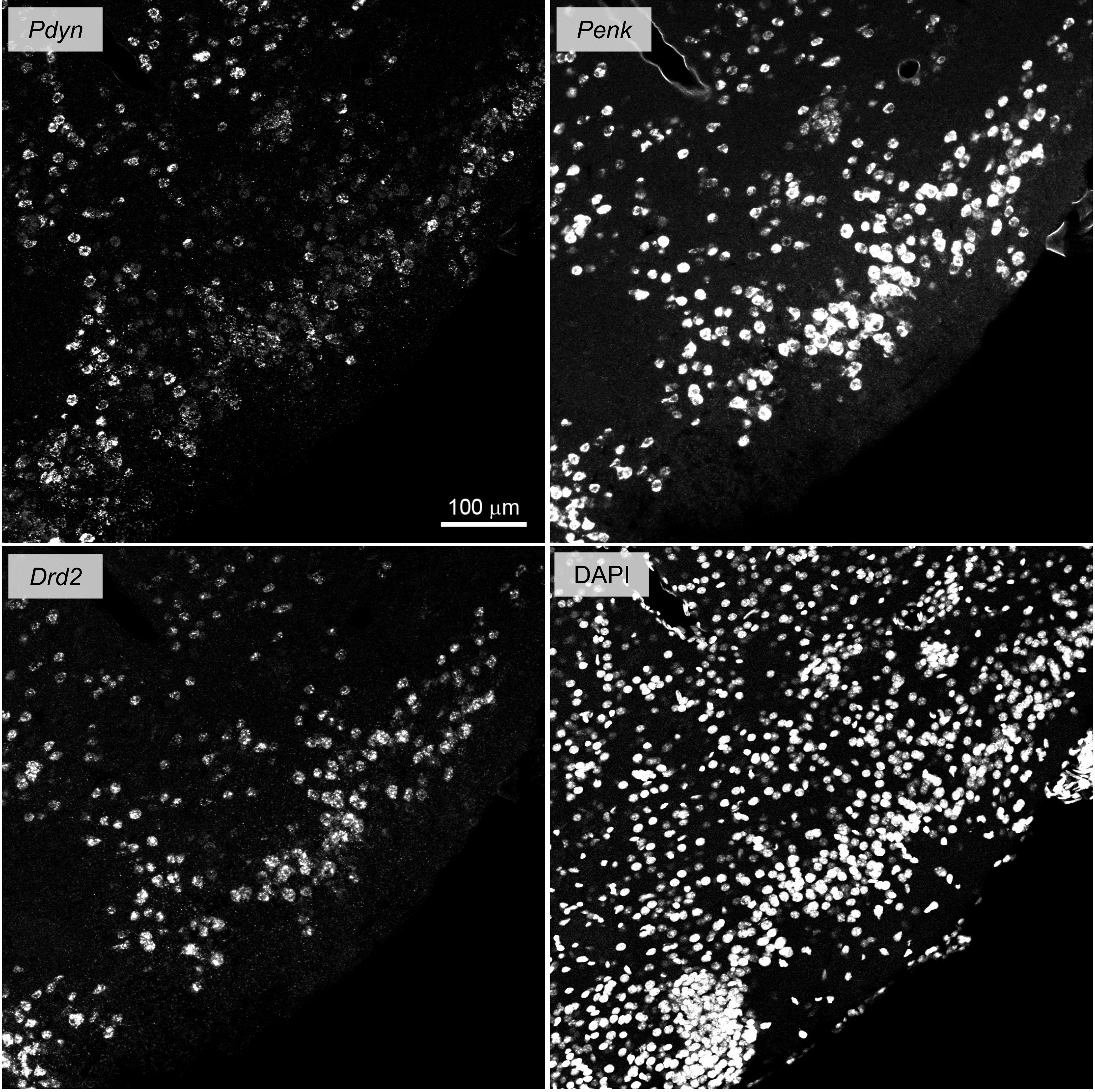

Supplementary Figure 3. DAPI(+) cells in the anteromedial DCL with triple fluorescence ISH signals of Pdyn-Penk-Drd2. Fig. 5 right panels were obtained by cropping these pictures.

Supplementary Table 1. Signal intensity of single ISH.

	Pdyn	*Penk*	*Drd1*	*Drd2*
MSNs in anteromedial DCL	+	++	+	+
MSNs in anteromedial DCL	+	++	+	+
dwarf cells, Cap	-*[1]	-	+	-
granule cells, Islands of Calleja	-	-	+*[2]	-

++; high intensity, +; moderate intensity, -; below detectable.
*[1]; faintly detectable, *[2]; detectable but low

Supplementary Table 2. The number of DAPI(+) cells examined and their colocalization of Pdyn-Penk-Drd1 and Pdyn-Penk-Drd2 signals.

Pdyn-Penk-Drd1 expression in the anteromedial DCL

	DAPI(+)	Pdyn(+)	Penk(+)	Drd1(+)	Pdyn(+) Penk(+)	Pdyn(+) Drd1(+)	Penk(+) Drd1(+)	Pdyn(+) Penk(+) Drd1(+)
mouse #1	381	121	124	170	31	118	37	30
mouse #2	317	115	128	146	37	107	37	37
mouse #3	347	129	150	144	35	110	43	35

Pdyn-Penk-Drd1 expression in the anterolateral DCL

	DAPI(+)	Pdyn(+)	Penk(+)	Drd1(+)	Pdyn(+) Penk(+)	Pdyn(+) Drd1(+)	Penk(+) Drd1(+)	Pdyn(+) Penk(+) Drd1(+)
mouse #1	360	115	123	153	24	88	21	18
mouse #2	370	96	150	157	9	77	9	6
mouse #3	348	105	128	153	17	77	19	15

Pdyn-Penk-Drd2 expression in the anteromedial DCL

	DAPI(+)	Pdyn(+)	Penk(+)	Drd2(+)	Pdyn(+) Penk(+)	Pdyn(+) Drd2(+)	Penk(+) Drd2(+)	Pdyn(+) Penk(+) Drd2(+)
mouse #1	380	122	145	138	37	11	109	11
mouse #2	349	105	113	105	32	16	99	15
mouse #3	437	134	166	130	45	7	116	7

Pdyn-Penk-Drd2 expression in the anterolateral DCL

	DAPI(+)	Pdyn(+)	Penk(+)	Drd2(+)	Pdyn(+) Penk(+)	Pdyn(+) Drd2(+)	Penk(+) Drd2(+)	Pdyn(+) Penk(+) Drd2(+)
mouse #1	377	95	148	145	18	15	135	14
mouse #2	354	98	136	137	24	14	136	14
mouse #3	393	116	143	141	17	7	126	4
